# Challenges faced by adolescents and lung function technicians in Anuradhapura district, Sri Lanka, during spirometry: a qualitative study

**DOI:** 10.1093/inthealth/ihaf097

**Published:** 2025-09-04

**Authors:** Devanji Perera, Gajaba Nayakarathne, Parami Chandrasiri, Kasuni Ananda, Hasara Nuwangi, Shashanka Rajapakse

**Affiliations:** Faculty of Medicine and Allied Sciences, Rajarata University of Sri Lanka, Saliyapura, Anuradhapura 50008, Sri Lanka; Faculty of Medicine and Allied Sciences, Rajarata University of Sri Lanka, Saliyapura, Anuradhapura 50008, Sri Lanka; Faculty of Medicine and Allied Sciences, Rajarata University of Sri Lanka, Saliyapura, Anuradhapura 50008, Sri Lanka; Faculty of Medicine and Allied Sciences, Rajarata University of Sri Lanka, Saliyapura, Anuradhapura 50008, Sri Lanka; Centre for Health Services Studies, University of Kent, Giles Ln, Canterbury CT2 7NZ, UK; Department of Physiology, Faculty of Medicine and Allied Sciences, Saliyapura, Anuradhapura, Sri Lanka; Graduate School of Cancer Science and Policy, National Cancer Center, 323 Ilsan-ro, Ilsandong-gu, Goyang-si, Gyeonggi-do 10408, Republic of Korea

**Keywords:** adolescents, asthma, lung function technicians, lung function testing, spirometry

## Abstract

**Background:**

Spirometry is the gold standard test for diagnosing airway disease. However, conducting spirometry in adolescents (13–14 y of age) is challenging because technicians should build a good rapport and maintain the technical rigour required in adult testing. This study investigated the challenges in spirometry assessment of adolescents with severe asthma.

**Methods:**

This was a qualitative descriptive study conducted in the Anuradhapura district, Sri Lanka from February to April 2023. Spirometry was conducted according to standard American Thoracic Society/European Respiratory Society guidelines. Three instructors with adequate knowledge but no prior experience conducted spirometry in adolescents, while two independent researchers observed the participants, followed by in-depth interviews. Data were collected until data saturation was achieved. Thematic analysis was used to analyse the experiences and perspectives of lung function technicians and participants.

**Results:**

Data saturation was achieved with 15 adolescents (7 male, 8 female). Two (13.3%) participants showed airway obstruction, while one (6.67%) had significant reversibility. Demonstration and simultaneous execution to train the respiratory manoeuvres improved the responses, but the test was tiring for the trainee instructor. Most adolescents found the procedure exhausting, and the trainee instructor's self-confidence improved the adolescents’ performance.

**Conclusions:**

The study underscores the need for specialized training required to conduct spirometry in adolescents and better awareness for adolescents and parents of lung function testing.

## Introduction

Asthma is a non-communicable chronic disease characterized by airway inflammation and airway hyperresponsiveness. It is the most common chronic disease affecting children and adolescents.^1^ The estimated global prevalence of asthma was 260 million in 2021.^2^ South Asia reports the highest prevalence of asthma (39.87 million).^3^ South Asia and Southeast Asia also reported the highest number of deaths due to asthma (232 190 and 72 060, respectively) and the highest number of disability-adjusted life years (DALYs) (6.91 million and 2.68 million, respectively).^3^ Although the prevalence of current asthma (symptoms of asthma within the last year) among 13- to 14-year-old adolescents was 11.0% in 2022,^4^ a significant proportion of adolescents with symptoms of severe asthma had not been diagnosed by a physician.^5^ The underdiagnosis of asthma can be attributed to several factors, including limited public awareness and understanding of the condition, inadequate access to healthcare services, overburdened health systems, insufficient availability of diagnostic tools like spirometry, a lack of knowledge among healthcare providers and the absence or poor enforcement of asthma management guidelines.^6^ In 2023, the prevalence of parent-reported physician-diagnosed asthma, severe asthma and poor control of asthma in adolescents in the Anuradhapura district, Sri Lanka was 19%, 15.3% and 29.9%, respectively.^7^ Parent-reported physician-diagnosed asthma is defined as asthma clinically diagnosed by a physician based on clinical history without spirometry evidence. Severe asthma was defined as having four or more wheezing episodes (recurrent wheeze), wheezing affecting speech or sleep disturbance due to wheezing for one or more nights per week in the preceding year.^8^ Poor control of asthma is defined as a score of ≤19 on a self-administered asthma control test.^9^ Considering the high disease burden, early and objective diagnosis of asthma in children and adolescents is critical to enable proper asthma care to prevent irreversible consequences such as airway remodelling leading to permanent airway obstruction.^10,11^

Spirometry is the gold standard lung function test to diagnose asthma. Despite adolescents being capable of performing spirometry reliably, lung function testing of adolescents poses a different set of challenges compared with adults because children and adolescents may struggle with comprehending or executing complex breathing instructions required for accurate spirometry.^12^ Poor effort, especially during maximal inhalation, maximal exhalation and sustained exhalation, can affect the quality of the spirometry curves and result in the underdiagnosis of asthma among adolescents.^13^ The main challenges faced by lung function technicians when instructing children and adolescents to perform spirometry are obtaining maximal reproducible manoeuvres, detecting subject warning signs and a lack of previous experiences and skills.^14^ Inadequate training of lung function technicians leads to poor-quality spirometry test results.^15^ The optimisation of the interaction between participant and technician is important to obtain maximum effort from the adolescent while performing spirometry, thus the quality of the test in adolescents depends mostly on the motivation provided by the lung function technician.^14^

Globally, qualitative studies exploring the challenges faced by adolescents with symptoms of asthma and by lung function technicians during spirometry are scarce. We conducted a qualitative study using a simple random sample of 13- to 14-year-old adolescents with severe asthma identified in a recently published study who were selected during an asymptomatic period.^7,16^ The main objective of this study was to explore the challenges faced by both lung function technicians and adolescents during spirometry, including understanding the experiences and perspectives of lung function technicians and adolescents with symptoms of severe asthma.

## Methods

### Study design

This was a qualitative descriptive study conducted in the Anuradhapura district, Sri Lanka, from February to April 2023.

### Study participants and participant selection

Adolescents (age 13–14 y) with symptoms of severe asthma and attending government schools in the Anuradhapura municipal council area, Anuradhapura, Sri Lanka, were identified using the International Study of Asthma and Allergy in Childhood questionnaire in a recently published study.^7,17^ From the identified participants, we selected potential participants for this study using a simple random sampling method. During the selection, adolescents with contraindications for spirometry (including acute nausea and vomiting; having undergone recent eye, thoracic or abdominal surgical procedures; and recent history of haemoptysis of unknown origin) were excluded from the study.^18^ We assessed asthma symptom control using the asthma control test.^9^ As recommended in the published literature on qualitative methods, we initially aimed for at least nine interviews.^19^ We developed preliminary codes and themes while interviews were ongoing. By the 13th interview, no new themes or codes were identified. However, we conducted two more interviews to confirm that data saturation had been reached.^20^ The lung function technicians were new to lung function testing in adolescents and received adequate instruction and training on performing spirometry with a Vyntus IOS (Vyaire Medical, Mettawa, IL, USA). We purposefully selected lung function technicians who were new to testing adolescents to identify the challenges faced by lung function technicians when they start testing adolescents.

### Study tools

#### Asthma control test

The asthma control test is a self-administered tool that consists of five items used to identify adolescents with poorly controlled (score ≤19) and well-controlled asthma.^9^ The translated and validated versions of the local languages used in Sri Lanka were used in this study.^7^

#### Spirometry test

Spirometry testing was conducted by three lung function technicians new to testing adolescents. Before performing spirometry, height and weight were measured using a portable Stadiometer (Seca 213) and a digital bathroom scale (Seca Clara 803) according to standard techniques. Spirometry was conducted using the Vyntus IOS with MicroGrad bacterial/viral filter. Calibration of the equipment was performed according to the manufacturer's instructions on-site.

The adolescents were required to sit or stand wearing a nasal clip. Since the adolescents had no prior experience with spirometry, each adolescent was individually instructed and guided before and during spirometry to obtain a reproducible, valid flow-volume curve. Each adolescent held a disposable tube in his/her mouth in a manner to prevent leakage of air, wore a nasal clip to occlude the nose and performed the learned manoeuvre in the standing or sitting position with monitoring of the flow-volume curve on the computer screen in every attempt. A minimum of three attempts was recorded. Bronchodilator reversibility was demonstrated by administering a short-acting inhaled bronchodilator (200 μg of metered-dose inhaler salbutamol with a spacer device) and repeating spirometry testing after 15 min. The positive bronchodilator reversibility cut-off was >12% and a >200 ml improvement of forced expiratory volume in the first second (FEV_1_) from the baseline.^21^ Participants and their parents were informed to withhold inhalers as recommended by the Global Initiative for Asthma (GINA) guidelines.^1^

#### Spirometry data analysis

Flow-volume curves that demonstrated a rapid increase to the peak expiratory flow and a slow, steady decline were selected. The flow-volume curves that fulfil the acceptability and usability criteria were used for assessment.^18^ The volume scale was at 10 mm/l, the time scale was 20 mm/s and at least 1 s before the start of expiration was displayed in the flow-volume curve. For the flow-volume curve, the flow display was calibrated to 5 mm/l/s and the flow:volume ratio was 2 l/s:1 l.^18^ The quality categories A, B, C, D, E, U and F for forced vital capacity (FVC) and FEV_1_ were used in this study.^18^

The best FVC and the best FEV_1_ values were obtained by observing all the acceptable curves or by observing the usable curves if none were acceptable.^18^ Those values were obtained separately for the sets of pre-bronchodilator and post-bronchodilator manoeuvres. Reference values for Southeast Asians, as recommended by the manufacturer, and a lower limit of normal were used for this research. An FEV_1_:FVC ratio <0.8 was considered an obstructive airway disease, and if the post-bronchodilator improvement of FEV1 was >12% it was considered reversible. Participants who had an FEV_1_:FVC ratio ≥0.8 and an FVC% <80% were considered to have restrictive airway disease. Pseudo-restriction in spirometry refers to a pattern that mimics a restrictive lung defect (i.e. reduced FVC) but is not due to intrinsic lung restriction. Instead, it is typically due to air trapping from obstructive diseases like chronic obstructive pulmonary disease or asthma.^21^

#### In-depth interviews and participant observation

Each participant was observed by at least two investigators and independent field notes were written to understand the challenges faced by the participant as well as the lung function technicians while performing the lung function tests. The investigators focused on the reactions of both lung function technicians and adolescents, their interactions and behaviours during the test, the strategies employed by the technicians and the challenges faced by both. In-depth interviews were conducted on-site with each participant and with the lung function technicians following each test. This approach ensured capturing their experiences while the event was still recent, reducing the risk of losing nuanced details. The interviews were semi-structured, focusing on the challenges faced by the adolescents and technicians and their perspectives while allowing participants to elaborate on any aspect they deemed relevant to their experience with lung function testing. The interviews were recorded and transcribed by the research team. The duration of the interviews ranged from 30 to 40 min.

### Data analysis

The dataset consisted of 30 field notes, 15 transcripts of interviews with lung function technicians new to spirometry in adolescents and 15 transcripts of interviews with adolescents. The data collected were analysed using thematic analysis.^22^ Both interview transcripts and field notes were transcribed, coded and categorized into themes. Initially, two investigators independently coded the data of four interview transcripts manually and developed a coding scheme. The interview transcripts were kept in the original native language (Sinhala) to preserve meaning. The remaining interview transcripts and field notes were then coded using the developed coding scheme and new codes were added as necessary. After coding was complete, the subthemes and themes were identified inductively through multiple rounds of discussions among the research team until a consensus was reached. Two major themes were identified to highlight the perspectives and experiences of the lung function technicians and the adolescents, as they are the primary contributors to a successful spirometry test. For example, codes such as ‘perceptions about slow spirometry’ and ‘perceptions about forced spirometry’ were categorised under the subtheme ‘Executing lung function testing manoeuvres’, which is within the broader theme of ‘Experiences and perspectives of adolescents regarding spirometry’. We have also reported spirometry test results (Table 2 and Supplementary Information 1) to provide additional context about participants’ lung function.

### Ethics considerations

Ethical approval was obtained from the Ethics Review Committee, Faculty of Medicine and Allied Sciences, Rajarata University, Mihintale, Sri Lanka (ERC number ERC/2023/03). Parents and/or guardians were provided with participant information sheets and consent forms in the native languages (Sinhalese and Tamil) and informed written consent was obtained. Simultaneously, the adolescents were provided with assent forms in their native language, and written assent was obtained.

## Results

### Study participants

This qualitative study included 8 (53.3%) female and 7 (46.7%) male adolescents ages 13–14 y (Table 1). Their body mass index (BMI) ranged between 12 and 23 kg/m^2^, with six participants having a BMI <16 kg/m^2^, which could adversely affect their spirometry performance. Most participants (n=11 [73.3%]) had good asthma control. The three lung function technicians were all female and 23–25 y of age.

**Table 1.** Details of study participants (N=15)

Participant	Age (years)	Sex	Height (cm)	Weight (kg)	BMI (kg/m^2^)	Asthma control
1	13	Female	160	55	21	Poor
2	13	Female	146	26	12	Poor
3	13	Male	158	33	13	Good
4	13	Female	164	63	23	Good
5	13	Male	153	29	12	Good
6	13	Female	162	49	19	Good
7	13	Female	151	39	17	Good
8	14	Male	169	42	15	Poor
9	13	Female	145	37	18	Good
10	13	Female	143	38	19	Good
11	14	Male	163	48	18	Good
12	13	Male	157	37	15	Good
13	13	Male	149	51	23	Good
14	13	Male	161	45	17	Poor
15	13	Male	157	37	15	Good

### Spirometry results

**Figure 1.**
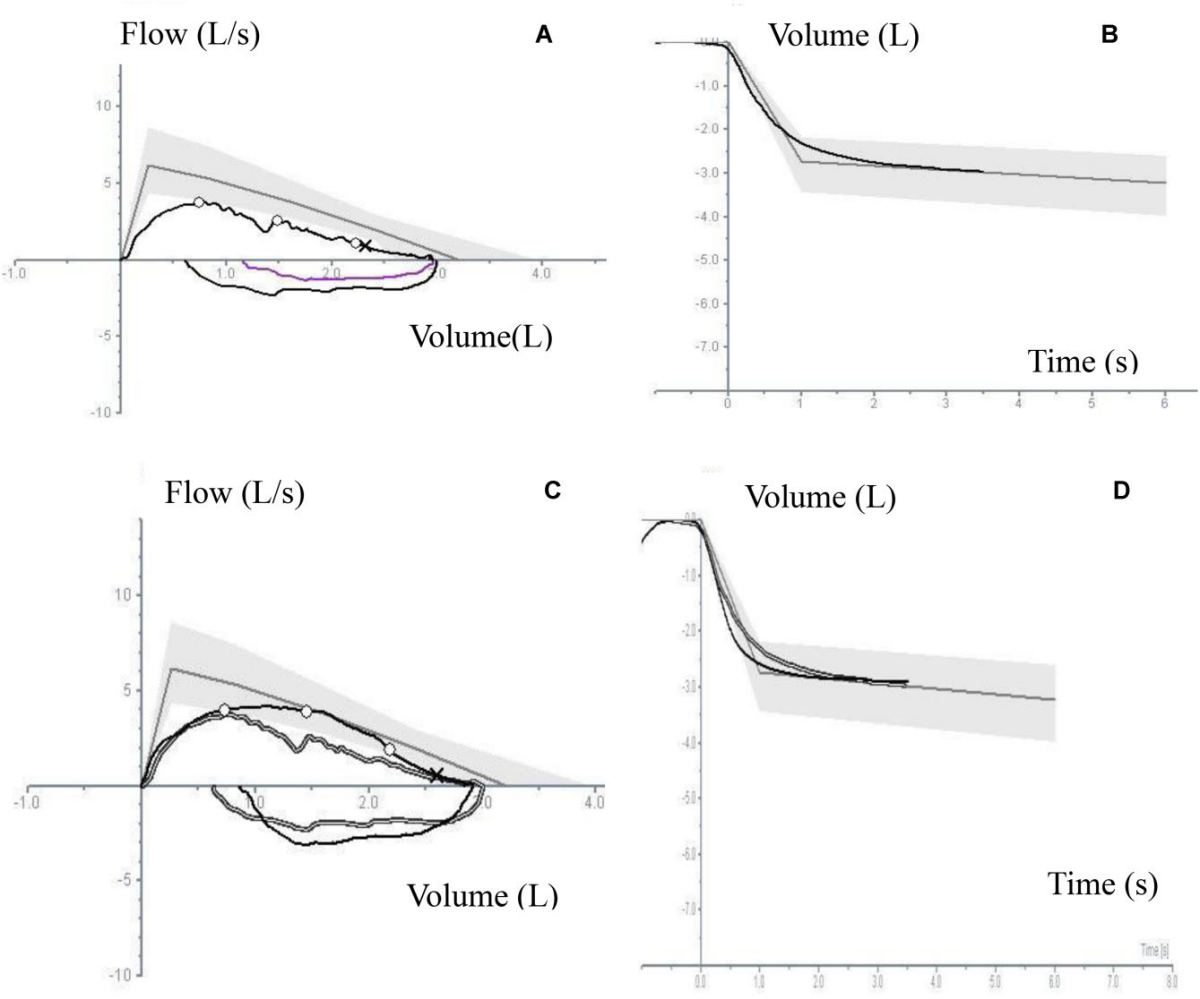
Pre-bronchodilator and post-bronchodilator administration forced spirometry–related graphs of participant 1 showing changes in flow-volume and volume-time curves. The post-bronchodilator response indicates a lack of significant reversibility. (**A)** Pre-bronchodilator flow-volume loop. **(B)** Pre-bronchodilator volume-time graph. **(C)** Post-bronchodilator flow-volume loop. **(D)** Post-bronchodilator volume-time graph.

The spirometry results of the research participants are provided in Table 2 and Supplementary Information 1. Airway restriction was noted in four participants, probably attributable to poor inspiratory effort (participants 2, 3, 4 and 8). Of the two participants who had airway obstruction, only one participant (participant 1) showed significant reversibility after administration of a bronchodilator, suggesting reversible airway obstruction (Figure 1). Participant 5, with pectus excavatum, had evidence of obstructive restrictive mixed airway disease. Three participants had reduced an FEV_1_:FVC ratio following the administration of β_2_-andrenergic agonists (participants 5, 9 and 14).

**Table 2.** Pre- and post-bronchodilator spirometry results of research participants (N=15)

												Grade		
Participant		FVC (l)	FEV_1_ (l/s)	FEV_1_%M (l/s)	FEV0.5 (l)	FEV0.5%M (l)	PEF (l/s)	MEF75 (l/s)	MEF50 (l/s)	MEF25 (l/s)	FET (s)	Pre	Post	Interpretation
1	Predicted	3.22	2.73	84.04			6.17	5.39	3.82	1.97		D	U	Obstruction
	Pre	2.97	2.31	77.15	1.58	52.6	3.81	3.71	2.53	1.04	3.49			
	%Predicted	92.23	84.61	91.8			61.75	68.83	66.23	119.8				
	Post	2.62	2.07	75.27	1.17	42.5	2.36	2.24	2.09	1.29	6.17			
	%Predicted	81.36	75.82	89.56			38.24	41.55	54.71	65.48				
	Change	−11.8	−10.4	−2.43	−26	−19	−25.8	−39.62	−17.4	24	76.7			
2	Predicted	2.49	2.13	86.41			4.98	4.42	3.13	1.61		F	F	Pseudo-restriction
	Pre	1.88	1.53	79.61	1.09	56.8	2.86	2.72	1.34	0.69	2.17			
	%Predicted	75.5	71.83	94			57.42	61.53	42.81	42.85				
	Post	1.89	1.77	94.04	1.35	71.4	3.44	3.29	2.7	1.24	11.73			
	%Predicted	75.9	83.09	111.1			69.07	74.43	86.26	77.01				
	Change	0.53	15.68	18.12	23.9	25.8	20.27	20.95	101.4	79.71	440.5			
3	Predicted	3.4	2.83	84.12			5.99	2.24	3.71	1.91		F	E	Pseudo-restriction
	Pre	2.35	2.12	89.23	1.32	55.5	2.64	2.05	2.38	2.3	2.37			
	%Predicted	69.11	74.91	106.1			44.07	39.12	64.15	120.4				
	Post	2.29	1.99	87.06	1.18	51.5	2.41	2.3	2.24	1.43	6.38			
	%Predicted	67.35	70.31	103.4			43.11	43.89	67.67	74.86				
	Change	−2.5	−6.13	−2.43	−11	−7.1	−8.71	12.19	−5.88	−37.8	169.2			
4	Predicted	3.45	2.92	83.87			6.54	5.68	4.03	2.08		F	F	Pseudo-restriction
	Pre	2.59	2.27	87.83	1.71	66	3.64	3.57	3.49	1.39	7.36			
	%Predicted	75.07	77.73	104.7			55.65	62.85	86.6	66.82				
	Post	2.59	2.35	90.87	1.91	73.3	4.75	3.5	4.26	1.53	7.41			
	%Predicted	75.07	80.47	108.3			72.62	61.61	105.7	73.55				
	Change	0	3.52	3.46	11.7	11.1	30.49	−1.96	22.06	10.07	0.67			
5	Predicted	3.09	2.58	84.33			5.56	4.89	3.46	1.78		E	E	Obstructive and restrictive
	Pre	2.17	1.71	78.96	1.31	60.4	4.04	3.93	1.77	0.65	6.26			
	%Predicted	70.22	66.27	93.63			72.66	80.36	51.15	36.51				
	Post	2.11	1.84	87.1	1.45	68.7	4.58	4.38	2.22	0.81	8.77			
	%Predicted	68.28	71.31	103.2			82.37	89.57	64.6	45.5				
	Change	−2.76	7.6	10.3	10.7	13.8	13.36	−11.45	25.42	24.6	40.09			
6	Predicted	3.33	2.83	83.96			6.35	5.35	3.92	2.02				Normal
	Pre	3.12	2.92	90.77	2.05	63.9	4.46	4.35	3.87	2.01	5.21	F	F	
	%Predicted	93.69	103.5	108.1			70.23	78.66	98.72	99.5				
	Post	3.13	2.9	89.36	1.92	59.1	4.12	4.03	3.61	2.41	10.24			
	%Predicted	93.99	102.5	106.4			64.88	72.87	92.09	119.3				
	Change	0.32	−0.76	−1.55	−6.3	−7.5	−7.62	−7.35	−6.71	19.9	96.54			
7	Predicted	2.73	2.33	84.41			5.39	4.76	3.37	1.73		F	E	Normal
	Pre	2.97	2.42	81.49	1.59	53.5	3.21	2.88	3.14	1.27	16.16			
	%Predicted	108.8	103.8	96.5			59.55	60.5	93.17	73.41				
	Post	2.87	2.47	86.03	1.47	51.2	3.01	2.91	2.88	1.88	22.55			
	%Predicted	105.1	106	101.9			55.84	61.13	85.45	108.6				
	Change	−3.36	2.06	1.92	−7.5	−4.3	−6.23	1.04	−8.28	41.73	39.54			
8	Predicted	4.14	3.43	83.67			7.01	6.06	4.3	2.22		F	B	Pseudo-restriction
	Pre	3.19	2.85	89.51	2.08	65.2	4.9	4.84	3.79	1.91	7.27			
	%Predicted	77.05	83.09	106.9			69.9	79.4	88.1	86.03				
	Post	3.32	2.9	87.54	2.3	69.3	6.8	6.67	3.96	1.37	12.42			
	%Predicted	80.1	85.5	104.6			97	110	92	61.7				
	Change	4.07	1.75	−2.25	10.5	6.24	38.77	37.8	4.48	−28.3	70.83			
9	Predicted	2.44	2.09	84.65			4.9	4.36	3.08	1.58		A	B	Normal
	Pre	2.06	1.4	69.37	0.99	38.1	2.07	2.03	1.29	0.27	14.11			
	%Predicted	84.4	66.9	81.94			42.24	46.55	41.88	17.08				
	Post	2.23	1.53	68.44	1.04	46.6	2.28	2.24	1.2	0.55	17.29			
	%Predicted	91.39	73.2	80.85			46.53	51.37	31.57	34.81				
	Change	8.25	9.28	−1.37	5.05	22.5	10.14	10.34	−6.97	103.7	22.53			
10	Predicted	2.34	2.01	84.74			4.74	4.23	2.99	1.53		B	F	Normal
	Pre	2.35	1.94	82.64	1.49	63.4	3.44	3.39	3.12	0.84	17.65			
	%Predicted	100	96	97.5			72.57	80.14	104.3	54.9				
	Post	2.12	1.88	88.08	1.38	64.5	2.81	2.26	2.8	1.34	5.89			
	%Predicted	90.59	93.53	103.9			59.28	53.42	93.64	87.58				
	Change	−9.78	−3.19	6.58	−7.4	1.7	−8.31	−33	−10.5	59.5	−66.6			
11	Predicted	3.73	3.09	83.91			6.44	5.61	3.98	2.05		U	U	Normal
	Pre	3.23	2.68	82.62	1.98	61.1	4.82	4.8	3.28	1.33	3.57			
	%Predicted	86.59	86.73	98.5			78.84	85.71	82.41	64.87				
	Post	3.17	2.73	86.09	2.07	65.3	5.09	4.97	3.58	1.46	15.74			
	%Predicted	84.98	88.34	102.6			79.03	88.59	89.94	71.21				
	Change	−1.85	1.86	4.19	4.54	6.84	5.6	3.54	9.14	9.77	340.8			
12	Predicted	2.44	2.09	84.65			4.9	4.36	3.08	1.58		F	A	Normal
	Pre	2.29	2.09	91.14	1.45	63.3	2.96	2.6	2.93	1.58	2.36			
	%Predicted	93.85	100	107.6			60.4	59.6	95.12	100				
	Post	2.56	2.13	82.81	1.71	66.8	4.26	4.12	3	1.24	24.02			
	%Predicted	104.9	101.9	97.8			86.9	94.4	97.4	78.48				
	Change	11.78	1.91	−9.13	17.9	5.49	43.91	58.46	2.38	−27.4	917			
13	Predicted	2.86	2.39	84.49			5.22	4.62	3.27	1.68		U	F	Normal
	Pre	2.81	2.34	82.6	1.71	60.2	3.92	3.89	2.87	1.24	12.39			
	%Predicted	98.25	97.9	97.76			75.09	84.19	87.7	73.8				
	Post	2.82	2.45	82	1.85	61.9	4.09	3.99	3.37	1.24	13.67			
	%Predicted	98.6	102.5	97.05			78.35	86.36	103.1	78.8				
	Change	0.35	4.7	−0.72	8.18	2.75	4.33	2.57	17.42	0	10.33			
14	Predicted	3.59	2.98	84			6.26	5.46	3.87	1.99		A	A	Normal
	Pre	2.79	2.33	86.8	1.67	59.9	4.26	4.02	2.67	1.2	19.01			
	%Predicted	77.71	78.18	103.3			68.05	73.62	68.99	60.3				
	Post	2.69	2.49	92.52	1.86	69.1	5	4.69	2.73	0.93	17.89			
	%Predicted	112.5	83.55	110.1			79.87	85.89	70.54	46.73				
	Change	−3.58	6.86	6.58	11.4	15.5	17.37	16.6	2.24	−22.5	−5.89			
15	Predicted	3.34	2.78	84.16			5.9	5.17	3.66	1.89		U	A	Normal
	Pre	2.52	2.22	87.32	1.62	63.8	3.79	3.69	2.86	1.23	7.58			
	%Predicted	75.44	79.85	103.8			64.23	71.37	78.14	65.07				
	Post	2.66	2.35	93.02	1.92	72.2	5.75	5.5	3.43	1.53	20.94			
	%Predicted	79.64	84.5	110.5			97.45	106.4	93.71	80.95				
	Change	5.55	5.85	6.52	18.5	13.2	51.71	49.05	19.93	24.39	176.2			

%Predicted: percentage predicted; Pre: pre-bronchodilator administration; Post: post-bronchodilator administration; %Change: percentage change between pre- and post-bronchodilator administration; FVC: forced vital capacity; FEV_1_: forced expiratory volume in the first second; FEV_1_%M: forced expiration volume in the first second as a percentage of maximal vital capacity; FEV0.5: forced expiratory volume in 0.5 s; FEV0.5%M: forced expiratory volume in 0.5 s as a percentage of maximal vital capacity; PEF: peak expiratory flow rate; MEF75: maximum expiratory flow rate at 75% of FVC; MEF50: maximum expiratory flow rate at 50% of FVC; MEF25: maximum expiratory flow rate at 25% of FVC; NR: not recorded.

### Thematic analysis

We explain our qualitative findings under two major themes: the experiences and perspectives of lung function technicians new to spirometry in adolescents and the experiences and perspectives of adolescents regarding spirometry.

### Experiences and perspectives of lung function technicians new to spirometry in adolescents

Field notes of observers from participant observation (n=30) and interview transcripts from in-depth interviews with lung function technicians (n=15) were analysed and the findings are explained under the following subthemes: instructions provided to the adolescent and the difficulties faced by technicians during each test.

#### Instructions provided to the adolescent

We observed that the lung function technicians gave verbal instructions, demonstrated respiratory manoeuvres and simultaneously executed breathing manoeuvres with the adolescent for training. They also used creative strategies, such as using party favours, to demonstrate the procedures.

All three technicians were confident that their subjects understood the verbal instructions. They also demonstrated the procedure of the test before the adolescents were tested, so that the adolescents understood the instructions better. Field note observations recorded that demonstrating the breathing manoeuvres helped the adolescents become familiar with the procedure and they appeared more relaxed.

Yes. The participant seemed to understand them (verbal instructions) well, but sometimes I was not quite sure…I demonstrated to the participant how to inhale, exhale, and blow as fast as possible…I think they understood better. (Technician 2c)

In certain instances, the lung function technicians synchronised their breath with the adolescent to obtain a better performance. This was challenging, as it made the process more exhausting for the lung function technicians.

An extract from the field notes stated:

The adolescent took small breaths during inhalation in the first attempt. Before the second attempt, the operator again demonstrated how they should exhale fully and reassured that the adolescent could expire for a longer period. However, the first two attempts were unsuccessful as the adolescent started inhaling before completely exhaling. Also, when the adolescent was asked to take a few cycles of normal breathing, on-screen monitoring showed that the adolescent was breathing too fast. The technician again demonstrated the technique by asking the participant to breathe simultaneously with the technician for training. Thereafter, the adolescent was able to expire for more than the required minimum time and performed better technically. (GN's field notes)

#### Difficulties faced during slow spirometry and forced spirometry

We identified the physical effort required to conduct the test, the need for extensive practice and building a good rapport with the adolescent as key difficulties faced by trainee technicians. When conducting the test, technicians found obtaining a good reading in forced spirometry, especially the FEV in the first second, as the most difficult step (Figure 1).

Maintaining the exhalation for 6 seconds was not satisfactory initially. Then, with further instructions and demonstrations, the participant did it correctly, but the participant did not perform the forced exhalation part satisfactorily, no matter how much I instructed and demonstrated. (Technician 3A)

We also observed that it was challenging for the technicians to get the participant to maintain the exhalation for 6 s during spirometry. Technicians were exhausted towards the end of the testing. They mentioned that encouraging the adolescent to exhale as fast as possible and maintain the exhalation for >6 s was difficult.

Yes. Sometimes we had to encourage the adolescents to continue to maintain their exhalation up to 6 seconds. Having to repeatedly instruct and encourage the adolescent was exhausting. (Technician 2B)

The technicians struggled to gain the trust and cooperation of the adolescents. It was also challenging to obtain the required readings without making the adolescents tired or uncomfortable.

I'm concerned about the connection I built up with the adolescent. I need to obtain good results with a minimum acceptable number of tries not to make the adolescent tired. So, I'm worried whether I am friendly enough, am I too demanding, or is the adolescent afraid of me? (Technician 1B)

### Experiences and perspectives of adolescents regarding spirometry

Field notes from participant observation (n=30) and interview transcripts from in-depth interviews with adolescents (n=15) were analysed and the findings are explained under three subthemes: comprehending instructions, executing breathing manoeuvres and perspectives of the adolescents and the parents about lung function testing.

#### Comprehending instructions

All the adolescents stated that they understood verbal instructions, but some stated that instructions for performing spirometry were unclear.

Instructions for that part (forced expiration) were a bit unclear, but once explained with the use of a party favour, it was understandable. (Participant P10)

After verbal instructions, lung function technicians gave demonstrations for further explanation of deep inhalation, deep exhalation and forceful exhalation. All adolescents agreed that the lung function technicians breathing alongside them to demonstrate the optimum technique was helpful and effective in remembering and following instructions and provided more clarity than verbal instructions.

…It was really helpful as all I had to do was breathe with the operator rather than listening and following instructions. (Participant P05)

#### Executing lung function testing manoeuvres

The challenges experienced by adolescents during lung function testing are comprehensively summarized in Table 3, providing a clear overview of the issues identified in our study. Most adolescents (n=8) stated that the overall test was tiring. The technicians were attentive and aware of the fatigue experienced by the adolescents and facilitated breaks as needed. Although they still found deep inhalation and exhalation exhausting, adolescents mentioned that the short breaks helped ease their fatigue.

**Table 3.** Difficulties encountered in conducting spirometry assessment in a cohort of 13- to 14-year-old adolescents (N=15)

	Difficulties in performing	
Research participant	Slow spirometry	Forced spirometry
1	Poor inspiratory effort Easily distracted deviating from given instructions	Easily distracted deviating from given instructions Poor inspiratory effort
2	Forced exhalation interrupted by small mid-expiratory inspirations	Poor effort in forceful exhalation
3	Insufficient expiratory time	Forced exhalation interrupted by small mid-expiratory inspirations
4	Insufficient expiratory time	Poor posture Forced exhalation interrupted by small mid-expiratory inspirations
5	Poor inspiratory and expiratory effort Restless	Poor posture Early termination of exhalation
6	Cough during expiration	Adequate performance
7	Early termination of inhalation and exhalation	Poor effort in forceful expiration
8	Poor inspiratory effort	Poor effort in forceful expiration Early termination of exhalation
9	Insufficient expiratory time	Early termination of exhalation
10	Insufficient expiratory time	Poor effort in forceful exhalation
11	Insufficient expiratory time	Poor effort in forceful exhalation
12	Poor posture	Poor effort in forced
13	Insufficient deep inhalation	Insufficient forceful exhalation
14	Adequate performance	Poor effort in forceful exhalation
15	Insufficient expiratory time	Poor expiratory effort

The test was tiring, but I got adequate rest in between the attempts. So, when I was asked to perform after a small break, I did not need further resting. (Participant P12)

An extract from the field notes states:

After the second attempt in slow spirometry, the adolescent leaned back into the chair and looked back towards the parent to make eye contact and smiled. The technician commented on the graph obtained and advised the adolescent to keep exhaling until she asked to stop. Then, the technician asked the adolescent whether he was tired, and the adolescent agreed. After a few minutes of rest, the test was performed again. The participant took around three normal breaths and a deep exhalation followed by a deep inhalation. After observing the graph, the technician said that the adolescent could take a deeper breath in, which showed visible improvement in the next attempt. After the technician had confirmed that slow spirometry was over, before moving to forced spirometry, the technician allowed the adolescent to rest again. (DP's field notes)

A few participants performed better when their posture was changed from sitting to standing.

Of slow spirometry and forced spirometry, most adolescents stated that forced spirometry was the most difficult test.

The second test (forced spirometry)…breathing out (fast) in the first second was difficult. I could not breathe as fast as I could when I was asked to…I think because I had to breathe out as fast as possible and then keep breathing out for as long as I could. (Participant P10)

During the rest, the adolescent was instructed on forced spirometry. In the first attempt, the adolescent was asked to take three cycles of normal breathing followed by forceful expiration, but as the performance in the first second was poor, the operator showed the graph on the computer screen and explained again that adolescents can expire more forcefully. In the second attempt, although the peak was obtained, the adolescent voluntarily stopped expiration. He was asked to exhale continuously for as long as he could without terminating his effort. It was noted that the adolescent was trapping air inside the mouth and the mouth was moving along the mouthpiece, accompanied by excessive body movements during exhalation. (DP's field notes)

Adolescents had different opinions about which part of spirometry was the most challenging. Some reported exhaling for 6 s was difficult, while others stated that deep inhalation was difficult.

When asked to breathe out for more than 6 seconds, I was scared that no more air was left in my lungs…but after two attempts I got used to breathing out for 6 seconds continuously (after the operator reassured the participant could exhale for more than 6 seconds). (Participant P01)

We observed that during deep exhalation for 6 s, most (n=9) of the adolescents were not maintaining an erect posture and they were sitting unsteadily with hands positioned either between their legs or grabbing the chair to force themselves to exhale for at least 6 s. When assessing lung function before and after the bronchodilator administration, most of the participants stated it was easier to perform the post-bronchodilator testing because they were more familiar with the technique.

#### Perspectives of the adolescents and the parents about lung function testing

None of the parents or adolescents had heard about lung function testing before the test. When the parents were approached first to explain the procedure, we observed that they were reluctant to let their child take the test. However, when explained using more familiar and similar tests, the parents understood the importance of spirometry for their objective diagnosis of asthma. Most adolescents were also initially hesitant, as they perceived the test involved invasive procedures. However, once they understood they only had to breathe in and out through the machine, they were cooperative.

The adolescents did not face any difficulty in handling the instrument. A few stated that they were scared when they first saw the instrument, but we observed that they relaxed once the procedure was explained.

## Discussion

This study was conducted to assess the challenges and experiences of lung function technicians and adolescents during spirometry. Seven of fifteen adolescents performed spirometry in an acceptable and reproducible manner, with three satisfying the acceptability criteria and two only satisfying the usability criteria. A previous study conducted in the UK reported that 68% of the adolescents (n=47, ages 5–16 y) presenting with wheezing or asthma performed spirometry in an acceptable manner.^23^ The same study showed that adolescents unable to perform spirometry were younger than those who could produce acceptable results. However, previous studies have shown that obtaining valid and repeatable lung function results in some preschool children is possible, but the performance is highly inconsistent.^10^ Repeat spirometry is recommended in symptomatic participants with normal spirometry results to avoid false negatives and underdiagnosis.^24^ Despite employing standardized spirometry protocols in our study, some adolescents were unable to generate acceptable curves, which further highlights the need for qualitative exploration of challenges in conducting spirometry in adolescents.

Most adolescents (46.67%) stated that the test was exhausting. A study by the American Thoracic Society and European Respiratory Society reported that 26.3% of participants felt tired after the spirometry test.^25^ However, the fatigue rate in our study was nearly twice as high, possibly due to the additional attempts required to achieve acceptable test results. Obtaining a technically satisfactory FEV_1_ was the most difficult step for both adolescents and trainees in our study. However, in contrast, a previous study on spirometry reported that forced expiratory measurements are easy to perform and that the measurements carry significant prognostic information.^26^ This may be due to differences in participant ages and the experience levels of lung function technicians. Trainee lung function technicians must receive comprehensive guidance and specialised training to obtain technically standard results in adolescents.^18^

Our findings show that technicians had to use on-screen incentives, demonstrations using party favours and repeated attempts to improve the performance of adolescents, as obtaining maximal forceful inspiration and expiration was difficult. This aligns with the current GINA guidelines that state many adolescents can perform reproducible spirometry if coached by an experienced technician with visual incentives. However, as shown by this study, a lack of instructions given to the adolescent throughout the test to maintain the required posture could affect performance during spirometry. This finding reinforces the need for specialized training and experience required to perform lung function tests with adolescents.

Demonstrating the breathing pattern and simultaneous execution of the respiratory manoeuvres with the adolescent before the test improved the performance of the adolescents, although it was tiring for the trainee instructor. A previous study showed that being adequately prepared for the test and receiving a comprehensive explanation of the process beforehand is essential.^25^ However, in our study, adolescents found it more helpful when each test was explained at the start and each step was called out to them during the test. Therefore, we suggest that demonstrating respiratory manoeuvres and synchronised breathing with the adolescent during training is superior to verbal instructions before the test.

We suggest that the training of lung function technicians should emphasize effective instruction techniques, strategies to improve adolescent cooperation such as the use of onscreen graphics and party favours, a convenient and comfortable testing environment, adequate rest periods between tests and methods to reduce the fatigue of technicians, as it is critical to conduct technically standard spirometry to promote objective airway disease diagnosis in symptomatic adolescents.^27^ Additionally, the experience of lung function technicians who are new to testing adolescents will improve over time with adequate and comprehensive training, ensuring progressively higher quality and reliability in test administration. Increasing awareness among parents about spirometry could improve test participation. Furthermore, as many adolescents with symptoms of severe asthma had normal spirometry results, incorporating complementary diagnostic methods could improve asthma detection and reduce underdiagnosis.

Our study has several limitations. The focus on a single geographic area may affect the generalizability of the findings to the whole country. However, this study intended to provide rich, in-depth data within the specified context.^28^ The short-term intraindividual variability, for instance, the variations in lung function throughout the day and airway reactivity, could have affected our results. A single spirometry test performed during an asymptomatic period may not definitively rule out asthma in adolescents. Therefore, it is important to consider re-evaluation at a later time to capture any changes in respiratory status and improve diagnostic accuracy. Next, limited background knowledge regarding lung function testing in adolescents and their guardians could have limited the scope of their ideas on spirometry. Many of the participants in our study were unaware that bronchial asthma can be diagnosed with spirometry. These factors may have affected their engagement, cooperation and performance during the testing, as well as the overall reliability of the data collected. We attempted data triangulation by using two qualitative methods—interviews and participant observation—and by using multiple observers rather than a single observer.^29^ Thus we recommend future research should focus on three key areas: developing and evaluating standardised training for lung function technicians to improve the quality of spirometry in adolescents, evaluating the effectiveness of alternative lung function tests in diagnosing asthma in adolescents and assessing the impact of interventions on increasing awareness of parents and adolescents about spirometry.

## Conclusions

This study provides insights into the challenges of conducting spirometry administered by technicians new to lung function testing among adolescents with severe asthma. Lung function technicians reported that they faced difficulties such as physical exhaustion and establishing rapport with adolescents. Regarding the experiences of adolescents, we found that clear explanation reduced their anxiety and the procedure was exhaustive but not scary. Adolescents struggled with deep inhalation, sustaining exhalation for 6 s and coordinating the timing of breathing manoeuvres. They found that demonstrations coupled with verbal instructions were more helpful. The need for breaks during the procedure and adjustments in posture were also important findings. Overall, this study highlights the need for specialised training required to conduct spirometry in adolescents and better awareness for adolescents and parents regarding spirometry.

## Supplementary Material

ihaf097_Supplemental_File

## Data Availability

All the data are included in the article.
