# Supplementary material for: Challenges faced by adolescents and lung function technicians in Anuradhapura district, Sri Lanka, during spirometry: a qualitative study
Source: Int Health. 2025 Sep 4;18(2):282–93. doi: 10.1093/inthealth/ihaf097 (PMC13016647; doi:10.1093/inthealth/ihaf097)
Supplement: ihaf097_Supplemental_File [file ihaf097_supplemental_file.pdf]

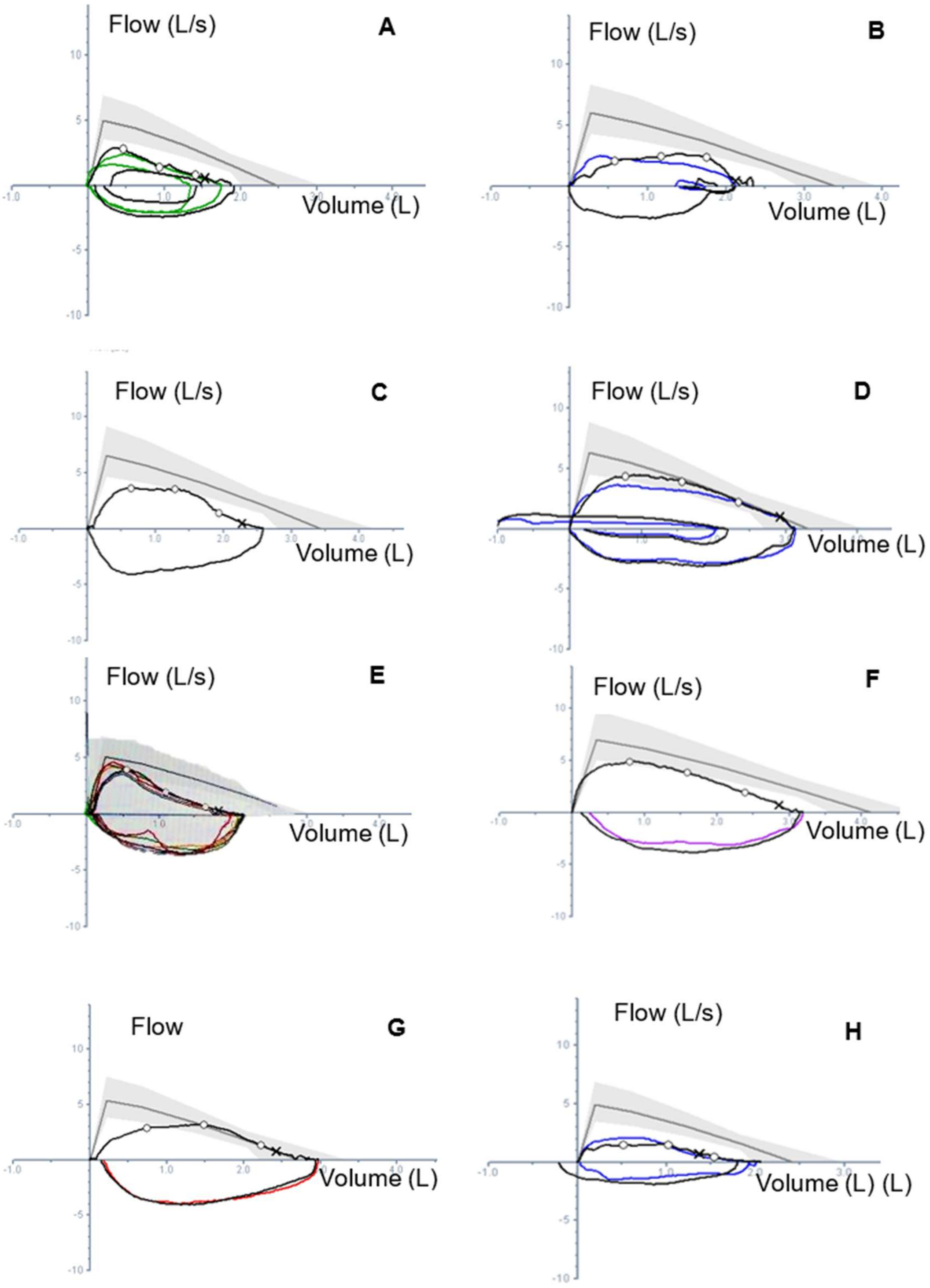

Flow (L/s)
A
Volume (L)
Flow (L/s)
B
Volume (L)
Flow (L/s)
C
Volume (L)
Flow (L/s)
D
Volume (L)
Flow (L/s)
E
Volume (L)
Flow (L/s)
F
Volume (L)
Flow
G
Volume (L)
Flow (L/s)
H
Volume (L) (L)

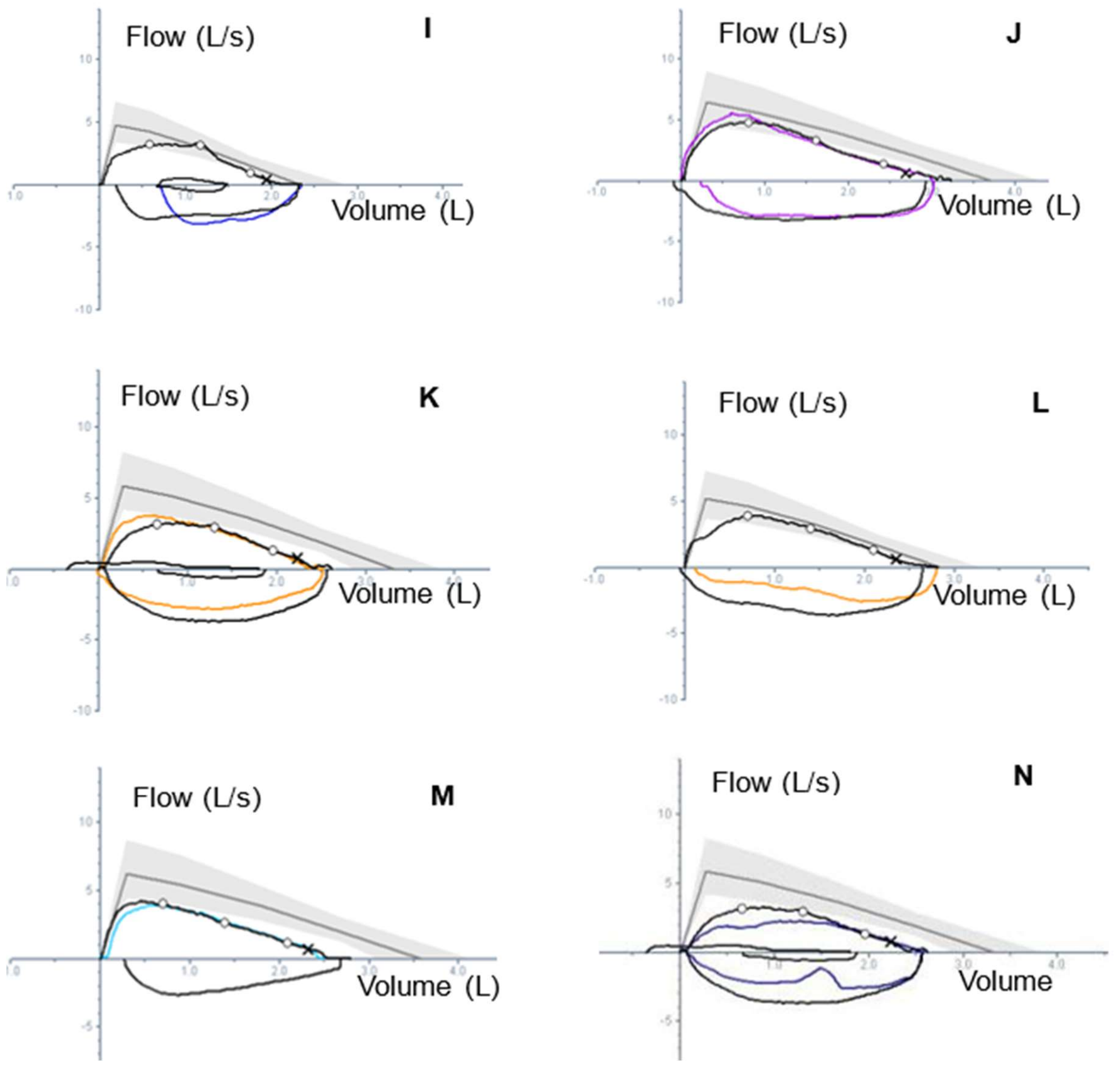

Supplementary Information 01: Pre-bronchodilator flow-volume forced spirometry graphs of participant 02 (A), participant 03 (B), participant 04 (C), participant 06 (D), participant 05 (E), participant 07 (F), participant 08 (G), participant 09 (H), participant 10 (I), participant 11 (J), participant 12 (K), participant 13 (L), participant 14 (M) and participant 15 (N)
